# Antibody-Dependent Enhancement: ″Evil″ Antibodies Favorable for Viral Infections

**DOI:** 10.3390/v14081739

**Published:** 2022-08-08

**Authors:** Xiaoke Yang, Xin Zhang, Xiaotian Zhao, Mengqi Yuan, Kehui Zhang, Jingwen Dai, Xiangyu Guan, Hua-Ji Qiu, Yongfeng Li

**Affiliations:** 1 State Key Laboratory of Veterinary Biotechnology, Harbin Veterinary Research Institute, Chinese Academy of Agricultural Sciences, Harbin 150069, China; 2 College of Animal Science and Veterinary Medicine, Henan Institute of Science and Technology, Xinxiang 453003, China; 3 College of Animal Science and Animal Medicine, Tianjin Agricultural University, Tianjin 300384, China

**Keywords:** antibody-dependent enhancement, Fc receptors, virus entry, immune responses, transcription modulation

## Abstract

The pandemics caused by emerging viruses such as severe acute respiratory syndrome coronavirus 2 result in severe disruptions to public health. Vaccines and antibody drugs play essential roles in the control and prevention of emerging infectious diseases. However, in contrast with the neutralizing antibodies (NAbs), sub- or non-NAbs may facilitate the virus to enter the cells and enhance viral infection, which is termed antibody-dependent enhancement (ADE). The ADE of most virus infections is mediated by the Fc receptors (FcRs) expressed on the myeloid cells, while others are developed by other mechanisms, such as complement receptor-mediated ADE. In this review, we comprehensively analyzed the characteristics of the viruses inducing FcRs-mediated ADE and the new molecular mechanisms of ADE involved in the virus entry, immune response, and transcription modulation, which will provide insights into viral pathogenicity and the development of safer vaccines and effective antibody drugs against the emerging viruses inducing ADE.

## 1. Introduction

Antibodies play an important role in the protective immune responses of host cells. As early as 2 to 3 days after onset of fever, virus-specific antibodies can be produced in plasma [1]. Neutralizing antibodies (NAbs) neutralize the incoming virus prior to virus entry into the host cell and do not affect viruses that have already infected cells. Luckily, Fc-mediated immune response can eliminate the virus-infected cells [2]. For example, non-NAbs mediate antibody-dependent cell-mediated cytotoxicity (ADCC) to clear virus-infected target cells [3,4]. For ADCC, virus-specific antibodies bind to the surface of the virus-infected cells and mediate cellular lysis by the activation of effector cells such as natural killer (NK) cells and monocytes. In addition, ADCC responses are required for immune protection against hepatitis C virus (HCV), herpes simplex virus (HSV), Epstein–Barr virus (EBV), influenza virus (IV), and human immunodeficiency virus (HIV) [3,4,5,6,7,8,9,10,11,12].

Compared with the neutralizing and ADCC antibodies, sub- or non-NAbs help virus to enter the target cells and promote viral infection, which is termed antibody-dependent enhancement (ADE) and was first reported by Hawkes in the 1960s. In cases of ADE, pre-existing sub- or non-NAbs facilitate viral entry into the target cells, leading to increased infectivity and pathogenicity rather than antiviral immunity. In the early study of ADE, it was demonstrated that the increase in viral production was caused by the combination of immune complexes and Fc receptors (FcRs); therefore, more infected cells were produced in the presence of antibodies than in the absence of antibodies [13,14,15,16]. For instance, the adhesion of West Nile virus (WNV) immune complex increased significantly compared with the naked virus particles in mouse macrophage-like cells [17,18]. Interestingly, it was found that the immune complex could inhibit the antiviral response of innate immunity by incubating the Ross River virus (RRV) with diluted anti-RRV serum, thus increasing the virus yield and enhancing the infectivity to cells. ADE is a complex phenomenon involved in the process of inhibiting innate immunity by immune complex [19]. The above discoveries fundamentally provide insights into ADE.

A number of viruses have been shown to be able to induce ADE, including *Flavivirus*: dengue virus (DENV), Zika virus (ZIKV), Japanese encephalitis virus (JEV), yellow fever virus (YFV), WNV, Murray Valley encephalitis virus (MVEV); *Coronavirus*: severe acute respiratory syndrome coronavirus (SARS-CoV), Middle East respiratory syndrome coronavirus (MERS-CoV), feline infectious peritonitis virus (FIPV); *Retrovirus*: equine infectious anemia virus (EIAV), HIV; *Arterivirus*: porcine reproductive and respiratory syndrome virus (PRRSV); *Pneumovirus*: respiratory syncytial virus (RSV); etc. [20]. Antibody-enhanced infection requires a “sensitized” initial immune event. At present, the following three cases have been reported: (1) naturally occurring initial infection with the same allotype virus, such as DENV; (2) chronic infections with viruses with antigenic diversity, such as lactate dehydrogenase-elevating virus (LDV) [21,22]; (3) antibodies from active or passive immunization that do not confer complete protection [21,22,23,24].

ADE is mediated by the FcRs on the cell surface [25]. Different types of FcRs play distinct roles in the pathogenicity of virus ADE. A variety of FcRs widely expressed on the surface of immune cells can specifically bind to Fc fragments of antibodies, promote the activation of immune cells, and trigger and regulate immune responses after the formation of Fc–FcR complex [26]. FcRs can be divided into Fc gamma receptors (Fc*γ*Rs), Fc epsilon receptors (Fc*ε*R), Fc alpha receptor (Fc*α*R), and Fc delta receptor (Fc*δ*R). Fc*γ*Rs play important roles in Fc–ADE, binding to IgG antibodies and mediating the interactions between immune cells and immune complex [27]. Three types of Fc*γ*R are present in humans: Fc*γ*RI (CD64), Fc*γ*RII (CD32), and Fc*γ*RIII (CD16). In addition, Fc*γ*RIV has been recognized in mice [28]. FcγRs can be divided into activated and inhibited types according to the signal transduction of the intracellular domain. The activated types of human Fc*γ*Rs include Fc*γ*RI, Fc*γ*RIIa, Fc*γ*RIIc, and Fc*γ*RIIIa, while Fc*γ*RIIb represents the sole inhibitory Fc*γ*R. Fc*γ*Rs are widely expressed on the surface of lymphocytes and myeloid cells. However, Fc*γ*RIIb is the only expressed FcR in B cells, and natural killer cells express only Fc*γ*RIIIa. Remarkably, Fc*ε*R1G is necessary for the activation of functional Fc*γ*RIa and Fc*γ*RIIIa [29]. In addition, the surface expression of Fc*γ*R is regulated by cytokines, with pro-inflammatory cytokines activating the expression of activated Fc*γ*Rs and anti-inflammatory signals enhancing Fc*γ*RIIb expression. The transcription levels of Fc*γ*RII and Fc*ε*RI were significantly upregulated under PRRSV-antibody complex infection [30].

Complement-mediated ADE (C-ADE) happens when the combination of virus and antibody forms an immune complex following complement activation, binds to complement to form a complex (virus-antibody-complement), and then enters the cell, enhancing infection via complement receptors on the cell surface. C-ADE was first proposed by Cardosa et al., who found that the infectivity of WNV to FcR-carrying P388D1 cells was enhanced in the presence of antiviral IgM. The complement type 3 receptor (CR3) specific monoclonal antibodies (MAbs) but not the FcR antibody could block this enhancement [31]. C1 binds to the Fc terminal of the antibody and activates the classical complement pathway, leading to the activation of C3, which covalently binds to the antibody or virion. The antigen–antibody–complement complex binds to CRs on the cell surface via a C3 fragment, allowing the virus to readily adsorb to the cell surface. Another pathway is that the C1q interacts directly with viral proteins and C1qR on the cell surface.

Not only the cellular entry of the immune complexes but the extracellular complexes (virus–antibody–complement) can also activate complement pathways through deposition in airway tissue, which further induces the recruitment and activation of neutrophils, monocytes, and eosinophils and stimulates the production of a number of pro-inflammatory cytokines [32]. These cytokines trigger and maintain inflammatory processes and the innate immunity to fight against viruses. Nevertheless, unrestrained complement activation always contributes to disseminated intravascular coagulation (DIC), inflammation, cell death, and immune paralysis and ultimately leads to multiple organ failure and death [33,34].

## 2. Common Characteristics of the Viruses That Are Able to Induce ADE

ADE effect mostly occurs in mononuclear macrophages, such as monocytes, tissue macrophages, and dendritic cells. For example, macrophages are the main target cells of FIPV infection, and PRRSV infects porcine alveolar macrophages. DENV infects the mononuclear macrophages, and the sub- or non-NAbs mediate viral entry into the target cells. RSV infection may be increased by modifying antigen presentation to form non-NAbs in the lung parenchymal epithelial cells. Two completely different examples of antibody-dependent virus immunopathology were unified by their interaction with the innate immune system. In both cases, the antiviral responses of the target cells or the APCs were inhibited [35]. In vitro studies show that human myeloid cells have different responses to ADE: ADE can be promoted only in the human monocytes, activated macrophages, and mature dendritic cells and not in immature dendritic cells. Although immature DENV particles may not infect the human myeloid cells, DENV infection occurs easily in the presence of anti-DENV antibodies [36,37].

The ADE of viral infection is mostly mediated by the structural proteins or the epitopes that induce the sub- or non-NAbs. PRRSV has antigenic variability and macrophage affinity, and the main structural proteins encoded by genomes are: GP3, GP4, GP5, M, and N. Current studies indicate that GP5 and N are responsible for the PRRSV ADE (Table 1). It has been shown that GP5 can induce NAbs, but there is high variability in immune-dominant non-neutralizing epitope A (aa 27–35) in the external structure domain of GP5, which completely covers neutralizing epitope B (aa 37–45), reduces the neutralizing effect, and produces ADE [38]. The N protein of PRRSV is highly conserved, and the antibodies against N are all non-NAbs.

Coronaviruses encode four main structural proteins: spike protein (S), envelope protein (E), membrane protein (M), and nucleocapsid protein (N). The S protein is the receptor binding site, cytolytic site, and main antigen site and plays a key role in the process of virus adsorption and invasion. When a virus particle invades, it first needs to be able to recognize the host cell. The virus binds to the host cell receptor through the S protein. Then, S is cleaved into S1 and S2 subunits. S1 is responsible for the recognition of the receptor, while S2 initiates the membrane fusion between the virus and the host, breaking through the membrane barrier and entering the cell. It has been reported that the receptor of MERS-CoV is dipeptidyl peptidase 4 (DPP4), which is expressed in T cells, lung, kidney, placenta, liver, skeletal muscle, heart, brain, endothelium, and pancreas [45]. SARS-CoV invades the target cells through the interaction between the S protein and the human angiotensin converting enzyme 2 (ACE2) receptor. SARS-CoV mainly infects ciliated bronchial epithelial cells and human lung type II epithelial cells. The S protein binding to the receptor activates the fusion mechanism in two ways: (1) Binding of the S protein to receptor can directly stimulate the fusion process, similar to HIV; (2) binding to the receptor, the virion forms endocytic vesicles that promote membrane fusion in an acidic environment. Porcine epidemic diarrhea virus (PEDV) depends on the porcine amino-peptidase N (pAPN) to enter target cells. It has reported that PEDV S protein expressed by the BAC-to-BAC Baculovirus was immunized and challenged in piglets, and disease enhancement was found, indicating that there may be epitopes on the S protein that enhance infection [54]. This conclusion was also confirmed in subsequent investigations. MAbs to the S1-A subdomain of PEDV S protein are neutralizing, while MAbs of the S protein to conformational epitopes in S1-0 and S1-BCD linear residues are enhancing [47,55].

The flaviviral genome encodes three structural proteins (capsid (C), pre-membrane (prM)/membrane (M), and envelope (E)) and seven non-structural proteins (NS1, NS2A, NS2B, NS3, NS4A, NS4B, and NS5). Virus invasion is mediated by the interaction of the E protein with host cell surface receptors. The E protein can interact with various host factors to promote virus adsorption, internalization, membrane fusion, and invasion. At present, the ADE of flaviviral infection is mainly associated with the E and prM proteins. NAbs targeting the viral E protein provide protection against flaviviral infection in vivo but may promote viral infection by ADE when antibodies are weakly neutralizing or in sub-neutralizing concentrations. MAbs against the E protein domain II could enhance the infection of DENV2 in K562 cells. It has been proved that replacing the histidine at the 244 position on the E protein with alanine could inhibit the binding between the signal peptide and the E protein to eliminate DENV ADE. Further experiments showed that changing amino acid H244 did not affect the synthesis of viral structural protein but could inhibit the packaging and release of viral particles. The anti-E and -prM MAbs were incubated with different concentrations of DENV, and it was found that the anti-E MAbs had obvious neutralization, while the anti-prM MAbs had a strong ADE effect and insignificant neutralization. After the completely immature virus was treated with anti-prM MAbs, the infectivity of the virus was significantly enhanced [56]. In addition, the DENV mutant lacking the lysine at position 90 was not able to induce the ADE. It has been indicated that the proteins of E and prM play important roles in the DENV ADE [56].

Cross-reactive antibodies can mediate the ADE of viruses belonging to the same genus, such as Flavivirus and Coronavirus. SARS-CoV-2 ADE could be driven by other coronavirus infection that produces sub- or non-NAbs. Genome-wide analysis showed that SARS-CoV-2 had 79% homology with SARS-CoV, 50% homology with MERS-CoV, and 87.99% homology with BAT-CoV. Since BAT-CoV had more homology with SARS-CoV-2, previous exposure to such viruses could cause ADE of SARS-CoV-2 infection [57,58,59,60]. It has been shown that the anti-E and -prM MAbs of JEV and WNV can also promote the infection of other flaviviruses in FcR-expressing cells [61,62]. For example, almost all anti-DENV antibodies can promote ZIKV ADE [63]. DENV or ZIKV infection can induce cross-reactive antibody responses. Cross-reactive non-NAbs recognize two main immune epitopes: One is the prM protein epitope, and the other is the fusion loop epitope (FLE) on the E protein [36,64,65]. DENV ADE was found to be weakened by replacing the DENV Pr4 gene with the JEV gene for chimeric DENV, and the 5th leucine, 6th leucine, 7th phenylalanine, and 16th cysteine of pr4 were the key sites responsible for DENV ADE, which provides a new reference for the development of attenuated vaccines [56,66]. In animal models, prior inoculation with DENV enhanced replication in ZIKV-infected pregnant mice, and significantly increased placental damage, fetal growth restriction, and fetal reabsorption [67,68]. ZIKV envelope contains E and membrane proteins, among which E-dimers are symmetrically arranged into an icosahedral structure. After entering the target cells, under the influence of the acidic pH of the endosome, the E protein undergoes irreversible conformational changes, and the E-dimers become monomers and then form trimers for membrane fusion [69,70].

Many effective vaccine strategies have been developed to eliminate viral ADE. At present, the ZIKV E-dimer-based subunit vaccine deleting the FLE of the prM and E proteins can produce dimer-specific antibodies and prevent ZIKV challenge during pregnancy. Importantly, the antibodies induced by ZIKV E-dimer immunization do not cross-react with DENV, which is a significant breakthrough for eliminating ZIKV ADE [41,71,72]. The substantial cross-reaction between DENV and ZIKV was explored using a group of human MAbs generated from DENV-infected people. It was found that most anti-DENV MAbs could bind to and enhance ZIKV infection but not neutralize ZIKV. However, the MAbs-recognizing E-dimer could effectively inhibit the ADE of ZIKV infection in vitro and in vivo [41,71,72]. Although there are 41 to 46% sequence differences between the E proteins of ZIKV and DENV, the similarity is sufficient for anti-DENV antibodies to cross-react with ZIKV and drive ADE [73]. A non-neutralizing epitope on ZIKV E domain III (DIII) surrounding residue at position 375 was identified. Therefore, to improve the efficacy of subunit vaccines, selected epitopes on DIII of the ZIKV E protein were shielded using nanopatterning combined with the site-specific incorporation of non-canonical amino acids and site-specific functionalization of the protein with polyethylene glycol [74]. Remarkably, a plant-produced vaccine based on DIII of the WNV E protein protects mice from the challenge of WNV without enhancing ZIKV or DENV infection [75].

Taken together, the virus that infects the mononuclear macrophages has great potential to induce ADE, which can be mediated by the main structural proteins or the epitopes on the proteins that induce the sub- or non-neutralizing or cross-reactive antibodies. Notably, the proteins responsible for ADE can induce both neutralizing and non-NAbs, which significantly affect the vaccine and drug development. Therefore, the above characteristics of the emerging viruses must be considerable to analyze whether the viruses can induce ADE. For example, current studies on African swine fever (ASF) vaccine candidates have found that inactivated vaccines have a risk of increasing the severity of the disease, and some subunit vaccines have been immunized to cause faster death in pigs [76,77]. African swine fever virus (ASFV) infects the mononuclear macrophages and induces non-NAbs, imply that the virus may induce ADE, which needs to be confirmed by experiments in the future.

## 3. Molecular Mechanisms Underlying the ADE of Viral Infections

### 3.1. Facilitating Virus Entry into the Target Cells with or without Viral Receptors

**Figure 1:**
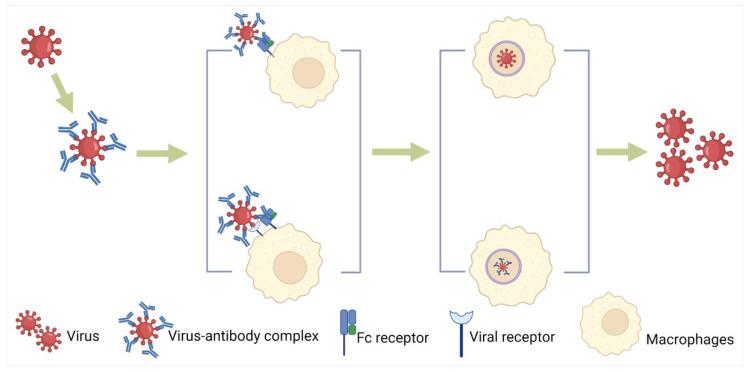
Facilitating virus entry into the target cells with or without viral receptors through endocytosis or micropinocytosis. There are two modes of FcR-mediated virus entry, one in which the virus–antibody complex only relies on the FcR on the surface of macrophages to mediate the endocytosis or micropinocytosis and one that requires the participation of the virus receptor.

Flaviviruses mainly rely on receptor-mediated endocytosis to enter the target cell. The entry of DENV into the target cell and subsequent early events of viral replication are key factors affecting the host response to infection. The ADE always takes place in a phagocytosis-like pathway by activating the cross-linking of Fc*γ*Rs on the surfaces of monocytes, macrophages, and dendritic cells (Figure 1) [78,79,80]. When there are antibodies with low neutralizing ability, the virus binds to the Fc fragment of the antibody and then promotes the adsorption and invasion of the virus under the mediation of Fc*γ*Rs (especially Fc*γ*RIIa and Fc*γ*RIIIa), thus enhancing the infection [42,50,73]. Non-NAbs against DENV promote the process of virus entry into monocytes and macrophages, particularly when the secondary infection has different DENV serotypes from the primary infection.

PRRSV binds to antibodies to produce antigen–antibody complexes, which bind to Fc receptors (mainly Fc*γ*R) on cell surfaces with the help of the Fc segment of the antibody and enter host cells through endocytosis. In addition, virus–antibody immune complex can enter the cell via micropinocytosis (Figure 1). When antibodies do not fully neutralize the virus, the virus enhances its susceptibility to host monocytes and macrophages through cell surface FcR (Fc*γ*RI/Fc*γ*RIIb/Fc*γ*RIII/Fc*ε*RI) mediation [81,82,83]. With Fc*γ*IR mediated, the expression of endocytic signaling pathway-related genes Akt and ERK1/2 was significantly increased, which promoted the formation and transport of mature lysosomes by promoting the expression and recruitment of microfilamentins, thus contributing to the entry of PRRSV/IgG immune complex into cells [81]. Fc*γ*R and CD_4_ are required for the ADE of HIV infection. In human primary macrophages, Fc*γ*RIII plays an important role in mediating HIV ADE. The binding of type 1 complement receptor (CR1) to CD4^+^ T lymphocytes enhances HIV replication in the target cells. It has been reported that IgA from HIV-1-infected patients mediated the infection enhancement in primary human monocytes with a macrophage-tropic strain of HIV-I [52]. Antibodies bind to HIV particles and adhere to the surface of target cells, promoting membrane fusion through FcR and CR to facilitate virus entry [52].

MERS-CoV and SARS-CoV rely on the receptor binding domain (RBD) of the S protein to combine with antibodies [84]. Low-titer RBD-specific MAbs increased the infectivity of SARS-CoV and promoted virus entry [45]. A recent study shows that the NAbs bound to RBD of MERS-CoV similar to the receptor (DPP4) binding and the former triggered ADE (Table 1). If MAbs do not trigger the conformational change of the coronavirus S proteins, neutralizing MAbs against other parts of coronavirus S protein are unlikely to mediate ADE. Therefore, in order to reduce ADE, a subunit vaccine based on the S protein lacking RBD can be designed to prevent viral infection. Based on the same principle, MAbs against other parts of the S protein can be selected to treat viral infection [85,86]. It has also been reported that using small S1 domain of MERS-CoV rather than the full-length spike protein was a better choice for developing a vaccine that eliminated the risk of ADE [87]. In vitro experiments, non-NAbs of the FIPV S protein, and highly diluted NAbs also enhanced FIPV infection in macrophages, and 50% of animals (cats) who have received passive immunization have peritonitis [88,89]. Generally, only FcR is able to mediate the entry of the viruses inducing ADE. However, ACE2 can act as the secondary receptor in the Fc*γ*R-dependent ADE of SARS-CoV-2 (Figure 1) [90].

Notably, the binding of MAb to viral protein can change the conformation of the ligands to facilitate viral entry. For example, MAbs targeting specific NTD sites could also induce conformational changes of the SARS-CoV-2 S protein RBD and enhance the binding ability of SARS-CoV-2 to ACE2 [91,92,93,94]. In addition, the same phenomenon has been observed for influenza virus. Using MAbs to analyze neutralizing or infection-enhancing epitopes on the hemagglutinin (HA), some MAbs neutralize virus infection at high concentrations of antibody and enhance virus infection at low concentrations [95]. Two murine MAbs (78/2 and 69/1) have been shown to increase IV fusion dynamics and promote IV infection in murine macrophage-like cell lines by disrupting the HA stem domain in vitro [53]. Therefore, ADE should be fully considered when treating patients with convalescent plasma or MAbs.

### 3.2. Changing the Innate Immune Response of Host Cells

**Figure 2:**
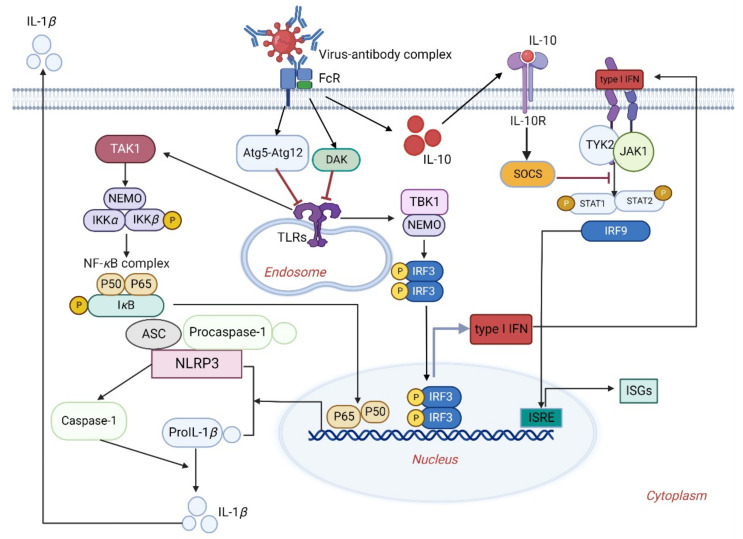
The distinct innate immune response of host cells under the ADE of viral infections. The virus–antibody complex binds to and activates FcRs that upregulate dihydroacetone kinase (DAK) and autophagy-related gene 5 and 12 (Atg5-Atg12 complex); it subsequently inhibits the TLRs activation and signaling pathway. Meanwhile, IL-10 activates the suppressor of cytokine signaling (SOCS) and then inhibits the Janus kinase-signal transducer and the activator of transcription (JAK-STAT)-specific pathway, resulting in the suppression of the interferon-mediated antiviral responses of host cells. On the other hand, release of the viral genome into the cytoplasm leads to the activation of TLRs, which subsequently increases IL-1*β* release by activating the NF-*κ*B signaling pathway.

It has been shown that both non-NAbs and highly diluted NAbs can increase the replication of FIPV in macrophages and enhance the production of inflammatory cytokines such as interleukin (IL)-1*β* (Figure 2) and IL-6 [46]. In addition, the expression levels of FIPV virus receptor (feline amino-peptidase N, fAPN) and TNF-*α* can be observed in macrophages inoculated with anti-S antibody and FIPV, which can further enhance the replication of FIPV in macrophages [46]. Although Fc*γ*RIIa and Fc*γ*RIIIa mediate the ADE of SARS-CoV-2 infection in vitro, the ADE effect may not be involved in aberrant cytokine release in macrophages during SARS-CoV-2 infection [96]. The ADE of DENV infection upregulates the production of anti-inflammatory cytokines but suppresses anti-DENV free radical and pro-inflammatory cytokine production in THP-1 cells. Collectively, the distinct levels of cytokine release can be induced by viruses under the ADE condition.

The molecular mechanism of PRRSV enhancing infection through ADE: The Fc*γ*Rs-mediated ADE plays a key role in the pathogenicity of PRRSV, and Fc*γ*RIIb is the only inhibitory receptor that interacts with PRRSV immune complex. It can prevent immune cells from becoming excessive by recruiting tyrosine inositol phosphate 1 (SHIP1) containing SH2 into cytoplasmic immunoreceptor tyrosine-based inhibition motif (ITIM) and inhibiting cytoplasmic signal transduction (TANK-binding kinase 1, interferon regulatory factor 3, and TBK-1-IRF3-IFN-*β*) [97,98]. In Fc*γ*RI-mediated PRRSV ADE, IFN production is inhibited by inducing the expression of pro-inflammatory cytokines, thereby inhibiting the immune responses [30]. In addition, the immune complex could downregulate the transcription levels of IFN-*α*, IFN-*β* and TNF-*α* and upregulate the expression levels of IL-10 under the mediation of porcine Fc*γ*RII and Fc*γ*RIII, thereby promoting the replication of PRRSV in porcine alveolar macrophages [51,99].

DENV infection under ADE not only promotes virus to enter the target cell but also inhibits the generation of type I IFNs, thus enhancing the replication in the host cells. In the model of ADE infecting THP-1 cells in vitro, DENV increased the infection through the specific Fc*γ*R pathway [100,101]. Sub-neutralizing antibody produced by DENV infection bound to Fc*γ*R, which could inhibit innate immune response through two pathways (Figure 2): (1) The upregulation of negative regulatory factors dihydroacetone kinase (DAK) and autophagy-related gene 5 and 12 (Atg5-Atg12 complex), which are recognized by pathogen-associated molecular patterns (PAMPs), can inhibit the expression of RIG-I like receptors (RLRs), Toll-like receptors (TLRs) and signaling pathway, thus inhibiting the innate immune response [35]; (2) through the early activation of IL-10. IL-10 activates the suppressor of cytokine signaling (SOCS), impairing Janus kinase-signal transducer and the activator of transcription (JAK-STAT)-specific pathway; it then inhibits type I IFNs signaling pathway and innate immune response. These two circular pathways inhibit the antiviral response of DENV-infected cells under ADE, resulting in the generation of a large number of infectious viruses [102]. Additionally, researchers used gene chip technology to analyze and compare the peripheral blood mononuclear cells (PBMCs) of children with DF and dengue hemorrhagic fever (DHF) at acute stage. The results showed that compared with patients with mild illness, the transcription levels of type I IFNs and IL-10 in the PBMCs of DHF patients were decreased. This study confirmed in vivo that DENV infection inhibited the antiviral response under ADE condition [78,79,103].

### 3.3. Changing the Transcriptional Levels of Host Molecules Supporting Viral Replication

By measuring the viral load of primary monocytes, it was found that antibody-dependent and antibody-independent entry pathways of DENV induced significantly different transcription reactions. The invasion of antibody-dependent DENV upregulated the expression levels of virus-dependent host factors related to RNA splicing, mitochondrial respiratory chain complex, and vesicle transport and blocked the classical receptor-mediated endocytosis to downregulate ribosome genes, which led to increasing virus replication. Briefly, antibody-dependent DENV infection changed the transcriptional levels of the host molecules supporting viral replication [65]. Furthermore, the transcriptional levels of Fc*γ*RII, Fc*ε*RI, protein kinase B (PKB), extracellular regulated protein kinases (ERKs), and other anti-inflammatory signaling molecules were significantly increased in porcine immunized with PRRSV-antibodies complex [30,103]. In addition, pre-inoculation with a formalin-inactivated RSV vaccine or measles virus (MV) vaccine caused a more serious disease in the subsequent natural infections. Using formalin to destroy the epitopes of RSV or making incomplete activation of TLR of APCs leads to the production of unprotected antibodies. Furthermore, more low-affinity antibodies with secondary stimulation in RSV-primed Th2 cells resulted in immune complex depositing into affected tissue [25,104]. It is feasible to vaccinate infants with safe and effective RSV vaccine containing TLR agonists, which can produce NAbs and confer protection [35].

## 4. Challenges and Prospects of Viral ADE

Using conventional vaccine immunization to prevent and treat viral diseases with ADE is often ineffective and even causes some diseases to worsen. The ADE of viral infections has brought great challenges to the development of vaccines. The study of ADE still faces many challenges, such as the lack of suitable animal models. Humans, mosquitoes, and lower primates are the natural hosts of DENV, but only humans infected with DENV have clinical symptoms. The WHO recommends using primates to assess the neuropathic damage of the attenuated yellow fever vaccine. However, the development of an appropriate animal model is the first difficulty to be overcome as transient viremia can only occur in primates after infections. At present, AG129 mice with the absence of IFN receptors are commonly used as animal models for the study of ADE [35]. Although ADE has been reported in SARS-CoV- or MERS-CoV-infected patients, the Fc*γ*R of model animals is still quite different from that of human beings [85]. In vitro experiments and animal models cannot completely predict the risks of ADE. Antibodies have very different properties in animals that are not predictive of those in the human host because the effector functions of antibodies are altered by species-specific interactions between the antibody and immune cells. Another reason is that the mechanisms mediated by protective and potentially harmful antibodies are the same, and highly diluted NAbs may also mediate virus entry. Therefore, we still need to distinguish how the antiviral response is harmful to human beings before we can make further use of ADE mechanisms to formulate effective vaccine strategies. Notably, new knowledge about Fc effector functions has led to improved passive antibody therapies through Fc modifications that reduce or enhance interactions with Fc*γ*Rs, lengthen the half-life of the antibody, and potentially enhance antigen presentation to T cells, posing a so-called vaccinal effect.

At present, ADE has been found for more viruses, including DENV, SARS-CoV, and PRRSV, and most of them are realized under the mediation of various types of FcRs. ADE has caused great difficulties for the development of safer vaccines for these viruses. Therefore, it is necessary for the development of safer vaccines to understand the underlying mechanisms of ADE mediated by FcRs. Analyzing the viral proteins and epitopes responsible for ADE and further exploring the underlying mechanisms give a significant clue to the development of safer vaccines and antiviral drugs. For example, since the specific antibodies directed against ZIKV NS1 in humans mediate ADCC and ZIKV NS1 does not have the risk of mediating ADE, NS1 can be used to develop effective vaccines [105]. Based on the study of coronavirus ADE mechanism, we can design subunit vaccines lacking RBD of the S protein to control viral infections. However, for DENV, an effective vaccine must have lasting neutralizing activity against all four serotypes of DENV. While ADE has been well documented in vitro for a number of viruses, the relevance of in vitro ADE for human coronaviruses remains less clear.

We can attempt to develop effective vaccines by utilizing the antiviral mechanisms of innate immune function or limiting ADE occurrence in vivo. ASF, which is caused by ASFV, is a transboundary epidemic prevalent in many affected countries. Currently, there is no commercial vaccine available for ASF. It is speculated that ADE is involved in ASFV infection, which significantly impaired vaccine development [106]. Thus, dissecting the ASFV ADE mechanism will be helpful for the development of a safe and effective vaccine against ASF. Interestingly, we may try to mutate or remove the antibody Fc region (resulting in single-chain or single-domain antibodies) to block the interaction between the anti-DENV antibodies and immune cells in order to eliminate ADE in antibody therapy. Additionally, cell-penetrating antibodies, superantibodies, or transbodies that directly target the viral proteases in cells could be developed. Although ADE hinders the development of vaccines and antibody drugs, we can use preexisting antibodies to enhance the viral entry into host cells to increase virus titers in cell cultures. In the future, mechanisms of producing NAbs, the antibodies causing ADCC or ADE, will be desirable to investigate.

Currently, the pathogenesis of SARS-CoV-2 ADE has been confirmed in vitro, but SARS-CoV-2 ADE in vivo needs more evidence. It has been confirmed that SARS-CoV-2 is not susceptible to macrophages, and there seems no need to consider the ADE of SARS-CoV-2, but it is noteworthy that lung epithelial cells express high levels of Fc*γ*RIIa [107,108,109]. In addition, COVID-19 patients have been reported to have a strong IgG antibody response to the nucleocapsid protein, resulting in delays in virus clearance and an increased severity of infection [45]. Furthermore, it was found that patients with weaker antibody response to the nucleocapsid protein had an early clearance of SARS-CoV-2. Hence, antibodies against the nucleocapsid protein of SARS-CoV-2 may not be neutralizing. These findings also validate the hypothesis of SARS-CoV-2 ADE [86,109,110]. In order to avoid ADE risk in vaccine development, safe SARS-CoV-2 T-cell vaccines that are not dependent on antibodies can be developed.

In conclusion, ADE is a major concern involved in virus pathogenicity, safer vaccine development, and antibody drugs. This review summarized the mechanisms of viral ADE from three aspects: promotion of virus entry, alteration of the antiviral response, and regulation of transcriptional levels of host molecules to support viral replication in the target cells. In the context of the COVID-19 pandemic or other emerging diseases, our review provides insights into the investigation of the potential risks associated with ADE.

## Figures and Tables

**Table 1:** The mechanisms underlying the ADE of viral infection.

Viruses	Ig Types	Fc Receptors	Viral Proteins Responsible for ADE	Mechanisms Underlying the ADE	References
DENV	IgG	Fc*γ*RI/Fc*γ*RIIa/Fc*γ*RIIIa	prM and E proteins	Facilitating virus entry into target cells Inhibiting innate immunity Changing the transcriptional levels of host molecules	[39,40]
ZIKV	IgG	Fc*γ*R	prM and E proteins	Facilitating virus entry into target cells	[41,42]
WNV	IgM	Fc*μ*R/CR	prM and E proteins	Facilitating virus entry into target cells	[43,44]
MERS-CoV/SARS-CoV	IgG	Fc*γ*RIIa	S protein	Mimicking the viral receptor using the MAb against the S protein to mediate viral invasion	[45]
FIPV	IgG	Fc*γ*RI/Fc*γ*RII	S and M proteins	Enhancing the production of inflammatory cytokines, such as IL-1*β* and TNF-*α*	[46]
PEDV	IgG	FcR	S protein	Enhancing viral infection in target cells	[47,48]
RSV	IgG	Fc*γ*R	G and F proteins	Stimulating poor Toll-like receptor (TLR) and producing non-protective antibodies	[49]
PRRSV	IgG	Fc*γ*RI/Fc*γ*RIIb/Fc*γ*RIII/Fc*ε*RI	GP5 and N proteins	Inhibiting the antiviral responses of host cells	[50,51]
HIV	IgG/IgA	Fc*α*R/Fc*γ*RIII/CR	GP160 protein	Promoting membrane fusion through FcR and CR to facilitate virus entry	[52]
IV	IgG	FcR	HA protein	Increasing IV fusion dynamics and promoting IV infection	[53]

## Data Availability

All data generated or analyzed during this study are included in this published article.
